# Decrease of 5-hydroxymethylcytosine in hepatitis B virus-related hepatocellular carcinoma: A cross-sectional study

**DOI:** 10.1097/MD.0000000000033943

**Published:** 2023-06-02

**Authors:** Yansheng Jia, Zhiquan Liu, Muwei Dai, Junhua Feng, Lihong Ye, Haicong Zhang, Erhei Dai

**Affiliations:** a Division of Liver Diseases, The Fifth Hospital of Shijiazhuang, Hebei Medical University, Shijiazhuang, Hebei, China; b Department of Pathology, the Fifth Hospital of Shijiazhuang, Hebei Medical University, Shijiazhuang, Hebei, China; c The Forrth Hospital of Hebei Medical University, Shijiazhuang, China.

**Keywords:** 5-hydroxymethylcytosine (5-hmC), hepatocellular carcinoma (HCC), hepatitis B virus, isocitrate dehydrogenase (IDH), ten-eleven translocation (TET)

## Abstract

Many epigenetic studies had found the decrease of 5-hydroxymethylcytosine (5-hmC) in various tumor tissues. However, limited information is available for hepatitis B virus-related hepatocellular carcinoma (HBV-related HCC). The present study aimd to investigate whether the decrease also existed in tumor tissues of HBV-related HCC and, if possible, to disclose its mechanism. We used immunohistochemistry and Image Pro Plus 6.0 Image Analysis Software to quantify the expression of 5-hmC, 5-methylcytosine, 10-eleven translocation (TET), isocitrate dehydrogenase (IDH) in pathological sections of tumor tissues and its para cancerous tissues of 40 HBV-related HCC patients. Our results showed that 5-hmC was decreased while 5-methylcytosine was increased in tumor tissues. We also detected TET1 and IDH2 were decreased in the tumor tissues and the decrease were positively correlated with the 5-hmC. The results suggested that the deficiency of 5-hmC was an epigenetic characteristic of HBV-related HCC and was mainly caused by the decrease of TET1 and IDH2.

## 1. Introduction

Hepatocellular carcinoma (HCC) ranks 3rd in the mortality rate of malignant digestive tumors worldwide, and its morbidity rate is increasing.^[1]^ Epigenetics plays an important role in the early stage of hepatocellular carcinoma.^[2]^ Epigenetics refers to changes in gene expression based on changes in non-deoxyribonucleic acid (DNA) sequences. DNA methylation and demethylation are the most common epigenetic forms.^[3]^ Changes in DNA methylation, including hypomethylation of oncogenes and hypermethylation of cancer suppressor genes, play important roles in tumor development.^[4–6]^ In DNA methylation, cytosines are changed into 5-methylcytosines (5-mCs) via the action of DNA methyltransferase. The 10-eleven translocation (TET) proteins catalyze the oxidation of 5-mC to 5-hydroxymethylcytosine (5-hmC), which is a demethylation step.^[7–9]^ Isocitrate dehydrogenase (IDH) is a key enzyme in the tricarboxylic acid cycle that catalyzes the oxidative decarboxylation of isocitrate to produce α-ketoglutaric acid (α-KG). TET is an Fe (2+)- and α-KG-dependent dioxygenase that must bind to α-KG to perform its function.

Many studies had detected the 5-hmC deficiency in various tumors which could be a feature as an important epigenetic marker for tumors.^[10–13]^ Studies had revealed that the degree of 5-hmC deficiency was an independent unfavorable prognostic factor for malignant tumors.^[14,15]^ Studies also had found decreased levels of 5-hmC in HCC and suggested that the shortage of TET and IDH might cause the decrease of 5-hmC.^[14]^ Hepatitis B virus infection is the most common cause of HCC worldwide, especially in China. However, the level of 5-hmC and the status of the TET enzyme family as well as the IDH in hepatitis B virus (HBV)-related HCC tissue was never reported before. This study aimed to identify the levels of 5-hmC, 5-mC, TETl, TET2, TET3, IDH1, and IDH2 in tumor and the adjacent tissues, so as to explore whether the 5-hmC is decreased in HBV-related HCC tissues and, if possible, to explore the associated mechanism.

## 2. Methods

### 2.1. Study design

We designed this cross-sectional study to investigate the expression of 5-hmC, 5-mC, TET, IDH in pathological sections of tumor tissues and its para cancerous tissues of 40 HBV-related HCC patients to explore whether the expression lever of 5-hmC is decreased and the relationship of its decrease and the lever of TET and IDH. The present study was a retrospective study of medical records and achieved samples obtained from patients with written informed consent. The study was approved by the Ethics Committee of the Fifth Hospital of Shijiazhuang City (Approval number: 2020020). The patient’s personal information were anonymized in the study and all data files.

### 2.2. Patient population

Forty HCC tissues and its para cancerous tissues were collected from patients at The Fifth Hospital of Shijiazhuang city from March 2015 to January 2017. The inclusion criteria of the patients were as follows: Hepatitis B surface antigen was positive for at least 6 months and; HCC was confirmed by histopathology. The exclusion criteria were as follows: Coinfection with hepatitis A, C, D, E or human immunodeficiency virus; Alcoholic liver disease, nonalcoholic fatty liver disease, diabetes, drug-induced liver damage, autoimmune liver disease, and extrahepatic tumor; Complicated with genetic or metabolic liver disease, hyperbilirubinemia caused by other causes and cholestatic liver disease; and Patients with a history of radiotherapy and chemotherapy. Among the 40 HCC cases, 25 were male and 15 were female. The mean age was 55.09 ± 9.73 years.

### 2.3. Laboratory treatment

Continuous sections of 4-μm-thick paraffin-embedded tissues were prepared, dewaxed and hydrated stepwise by xylene and gradient ethanol. The samples were subjected to antigen retrieval by microwave heating in citrate buffer. After natural cooling, the samples were washed 3 times with phosphate-buffered saline (PBS) for 5 minutes each, followed by incubation with 3% hydrogen peroxide solution for 20 minutes to remove endogenous peroxidase activity. Next, the samples were washed 3 times with PBS and then were incubated overnight with the following antibodies: rabbit polyclonal anti-5-hmC antibody (Active Motif, USA; 1:2000), rat monoclonal anti-5-mC antibody (Active Motif, USA; 1:400), rabbit polyclonal TET1 resistance antibody (Active Motif, USA; 1:1000), rabbit polyclonal anti-TET2 antibody (Abcam, USA; 1:200), rabbit polyclonal anti-TET3 antibody (Boorson, China; 1:400). Thereafter, the samples were incubated in PBS immersion for 20 minutes and then were subjected to the diaminobenzidine color reaction (controlled under the microscope), hematoxylin complex staining, ethanol dehydration, until transparent and sealing with neutral gum.

### 2.4. Quantitative analysis

Positive expression was determined by the presence of brown-yellow granules in the nucleus/cytoplasm of hepatocytes or HCC. To carry out semiquantitative analysis more accurately, 5 fields were randomly selected from each section to be photographed under the microscope. All the photos were acquired under the settings of the same microscope and were analyzed using Image Pro Plus 6.0 (Media Cybernetic Inc., USA) image analysis software. For the immunohistochemical results of quantitative analysis, the positive research cell area (Area), average optical density value (mean density, MD) and integral optical density (IOD) value were measured as the fomula: IOD = Area × MD. Five sets of data were obtained from each section, and the average was used as an effective indicator for the quantitative analysis of immunohistochemical results. The methods adopted in this analysis were as follows: Open the Image Pro Plus 6.0 image analysis software and open the image: convert the optical density unit intensity format. Set the color selection parameter (after the parameter is set, all images will be determined under this parameter). Remove background. Select measurement values: area, density (mean) and IOD. In this experiment, due to the same photo area, the IOD value of the average of 5 sets of data was used for analysis.

### 2.5. Statistical methods

Solutions statistical Package for The Social Sciences 19.0 (SPSS Inc., USA) statistical software was usedfor analysis. The measurement data were expressed as the means ± standard deviation, and Student *t* test of the paired data was used for analysis. *P* < .05 was considered significantly different. The relationship between the factors were analyzed with Pearson Correlation test, and *P* < .05 was considered to be statistical significantly.

## 3. Results

**Figure 1.**
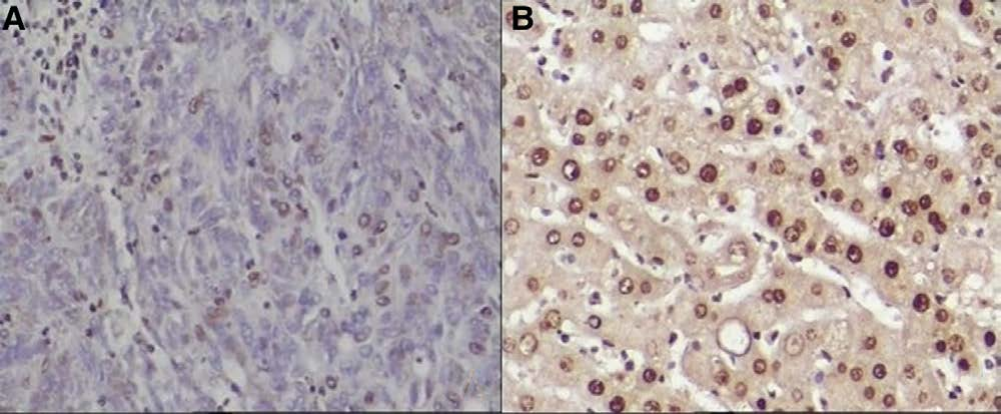
5-hmC expression in cancer tissues and adjacent tissues of HBV-related HCC patients (x200 stain: hematoxylin A: cancer tissue; B: paracancerous tissue). 5-hmC = 5-hydroxymethylcytosine, HCC = hepatocellular carcinoma.

**Figure 2.**
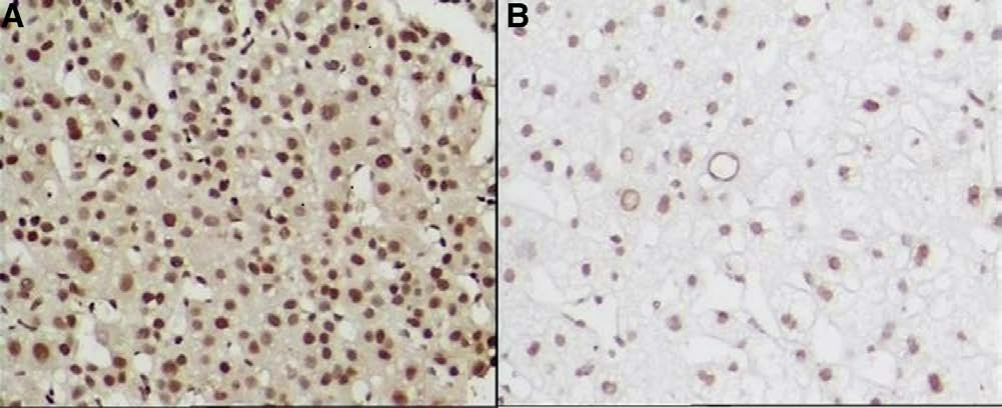
5-mC expression in cancer tissues and adjacent tissues of HBV-related HCC patients (x200 stain: hematoxylin A: cancer tissue; B: paracancerous tissue). 5-mC = 5-methylcytosine, HBV = hepatitis B virus, HCC = hepatocellular carcinoma.

**Figure 3.**
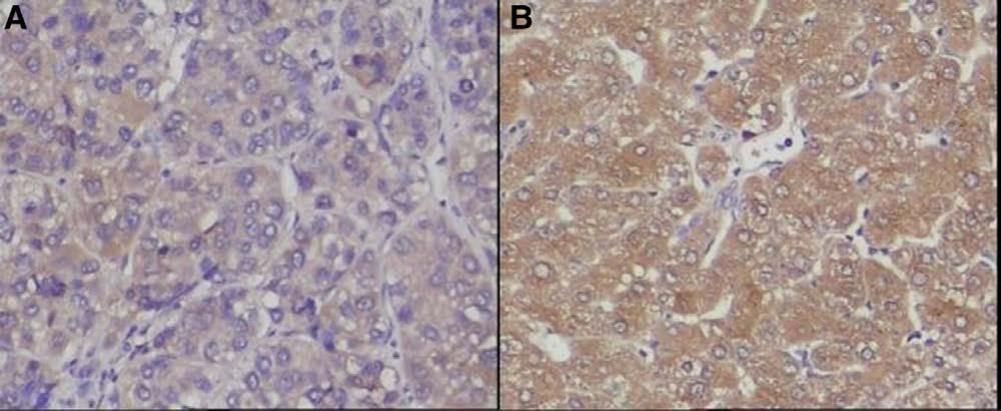
TET1 protein expression in cancer tissues and adjacent tissues of HBV-related HCC patients (x200 stain: hematoxylin A: cancer tissue; B: paracancerous tissue). HBV = hepatitis B virus, HCC = hepatocellular carcinoma, TET = ten-eleven translocation (TET).

**Figure 4.**
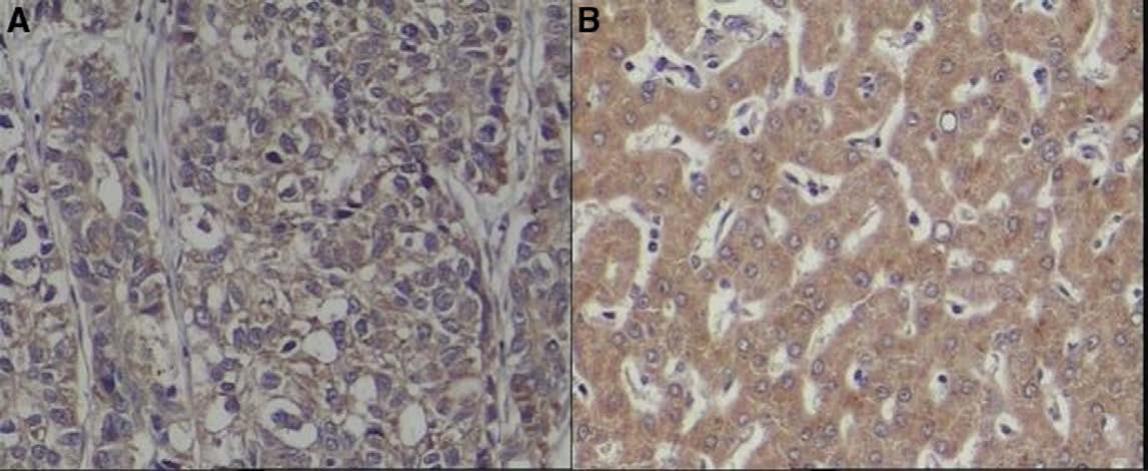
TET2 protein expression in cancer tissues and adjacent tissues of HBV-related HCC patients (x200 stain: hematoxylin A: cancer tissue; B: paracancerous tissue). HBV = hepatitis B virus, HCC = hepatocellular carcinoma, TET = ten-eleven translocation (TET).

**Figure 5.**
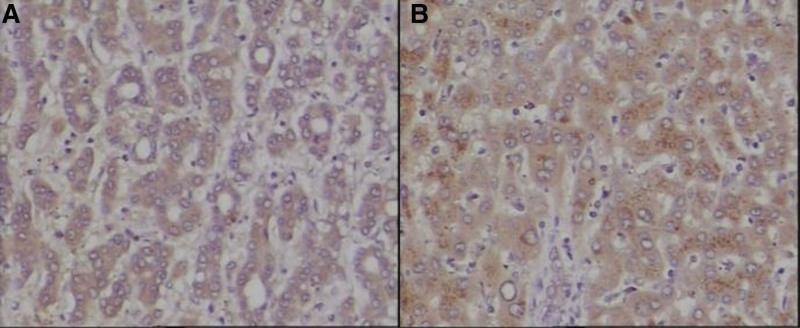
TET3 protein expression in cancer tissues and adjacent tissues of HBV-related HCC patients (x200 stain: hematoxylin A: cancer tissue; B: paracancerous tissue). HBV = hepatitis B virus, HCC = hepatocellular carcinoma, TET = ten-eleven translocation (TET).

**Figure 6.**
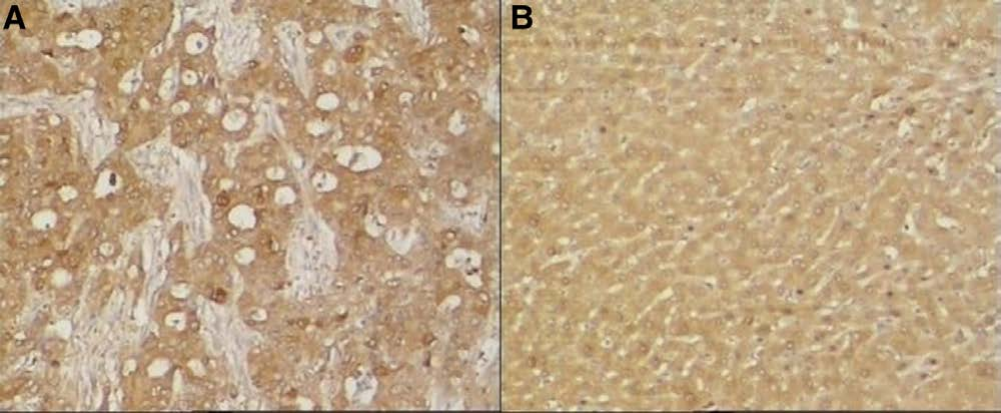
Expression of IDH1 protein in cancer tissues and adjacent tissues of HBV-related HCC patients (x200 stain: hematoxylin A: cancer tissue; B: paracancerous tissue). HBV = hepatitis B virus HCC = hepatocellular carcinoma, IDH = Isocitrate dehydrogenase.

**Figure 7.**
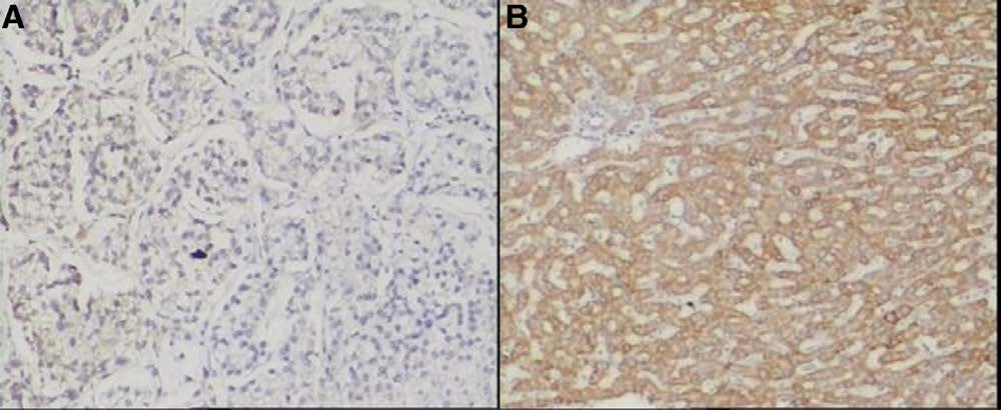
Expression of IDH2 protein in cancer tissues and adjacent tissues of HBV-related HCC patients (x200 stain: hematoxylin A: cancer tissue; B: paracancerous tissue). HBV = hepatitis B virus, HCC = hepatocellular carcinoma, IDH = Isocitrate dehydrogenase.

1. 5-hmC and 5-mC were mainly expressed in the nucleus of liver tissues. The IOD values of 5-hmC expression in the tumor tissues were lower, while the 5-mC were higher than the para cancerous tissues (*P* < .001). (See Figs. 1 and 2 and Table 1)
2. TET1, TET2, and TET3 proteins were mainly expressed in the cytoplasm of liver tissues. The IOD values of TET1, TET2, and TET3 protein expression levels in HCC tissues were significantly lower than those in adjacent tissues. (See Figs. 3–5 and Table 1)
3. The IDH1 and IDH2 proteins were mainly expressed in the cytoplasm of liver tissues. The IOD values of IDH1 and IDH2 suggest that there is no significant difference in the IDH1 protein expression between the HCC tissues and the adjacent tissues (*P* > .05). However, the expression level of IDH2 protein was significantly decreased in the tumor tissues (*P* < .001). (See Figs. 6 and 7 and Table 1)
4. The results of Pearson correlation analysis showed that 5-hmC was negatively correlated with 5-mC and positively correlated with TET1 and IDH2 in tumor tissue. There is also a positive correlation between TET1 and IDH2 in the tumor tissues of HBV-related HCC patients. (See Table 2)

**Table 1:** Results of Comparison of IDO values in tumor tissues and adjacent tissues for certain factors.

Variables	Patients number	Tumor tissue	Paracancerous tissue	*P* value
5-mC	40	70293.6 ± 55960.0	20505.4 ± 20223.2	*P* < .001
5-hmC	40	25240.5 ± 31297.6	51919.8 ± 52554.8	*P* < .001
TET1	40	13575.9 ± 44005.0	231986.7 ± 182114.3	*P* < .001
TET2	40	103115.1 ± 98528.1	303669.9 ± 111943.4	*P* < .001
TET3	40	76898.0 ± 98601.1	302471.2 ± 168723.4	*P* < .001
IDH1	40	317376.4 ± 117957.5	335776.6 ± 120881.7	*P* = .953
IDH2	40	74851.8.0 ± 95864.2	136895.1 ± 92902.4	*P* < .001

*P* < .01 means the difference of the two values is significant.

5-hmC = 5-hydroxymethylcytosine, 5-mC = 5-methylcytosine, IDH = Isocitrate dehydrogenase, TET = ten-eleven translocation (TET).

**Table 2:** Results of Pearson correlation analysis among the factors in tumor tissues.

Factors	5-mC	5-hmC	TET1	TET2	TET3	IDH1	IDH2
5-mC	1	−0.568*	−0.637*	−0.219	−0.337	−0.126	−0.713*
5-hmC	−0.568*	1	0.633*	0.346	0.482	0.249	0.599*
TET1	−0.637*	0.633*	1	0.159	−0.096	0.135	0.641*
TET2	−0.219	0.346	0.159	1	0.317	0.516*	0.558*
TET3	−0.337	0.482	−0.096	0.317	1	0.310	0.702*
IDH1	−0.126	0.249	0.135	0.516*	0.310	1	−0.336
IDH2	−0.713*	0.599*	0.641*	0.558*	0.702*	−0.336	1

5-hmC = 5-hydroxymethylcytosine, 5-mC = 5-methylcytosine, IDH = Isocitrate dehydrogenase, TET = ten-eleven translocation (TET).

* Significance test results (two tails) < 0.01, the correlation is significant.

## 4. Discussion

Methylation and demethylation in DNA epigenetics are persistent topics in cancer research. 5-hmC is an intermediate product in the cytosine methylation process. Studies had reported reduced 5-hmC levels in various tumors, which was an important epigenetic marker for tumors.^[10,15–19]^ However, no reports were found to investigate whether the 5-hmC level in HBV-related HCC tissues was also reduced as in other tumors. In our study, we detected the levels of 5-hmC in 40 cases of HBV-related HCC tissues and the corresponding tissues adjacent to the carcinoma. We found that the 5-hmC levels in HBV-related HCC tissue were significantly lower while the 5-mC levels were higher than those in tissues adjacent to the carcinoma. These findings were similar with the results of Chen et al.^[19]^

The TET enzyme plays a crucial role in DNA demethylation, catalyzing 5-mC to 5-hmC. Three TET proteins exist in mammalian cells, TETl, TET2 and TET3, which play different roles in different tissues. Researchers found that the TET proteins maintained a relative balance of 5-hmC and 5-mC levels. The balance was broken after TETl and TET2 were inhibited and caused changes in the expression of a series of genes.^[5,8,20–22]^ Chen and others found that the significantly reduced 5-hmC in HCC and other tumors was related to the downregulation of the TET enzyme family.^[19]^ Yang and others reported that the reduced 5-hmC level in human breast cancer and HCC tissues might due to the decreased expression levels of TETl, TET2, and TET3, with the most significant reduction of TETl.^[16]^ Sajadian found that the significant decline of 5-hmC in HCC and other tumors was related to the downregulation of TET2.^[23]^ Thus, controversy existed in regarding which member of the TET enzyme family played the most important role in reducing the 5-hmC level in HCC patients. In China, HBV infection is the main cause of HCC. Whether the mechanism of 5-hmC reduction in the tumor tissues of HBV-related HCC patients is also related to the downregulation of the TET enzyme family and which member of the TET enzyme family plays the key role in this process has not been reported. Therefore, we detected the levels of 5-mC, 5-hmC, TETl, TET2, and TET3 in tumor tissues and the corresponding para cancerous tissues of 40 patients with HBV-related HCC. We found the TETl, TET2, and TET3 protein levels in tumor tissues were significantly lower than those in para cancerous tissues, and the decrease of TET1 has a positive correlation with the decrease of 5-hmC in tumor tissues. These findings suggested the TET1 involved in the decrease of 5-hmC in tumor tissues of HBV-related HCC. Our findings were consistent with those reported by Liu, et al.^[24]^

IDH is an important enzyme in the tricarboxylic acid cycle, which catalyzes isocitrate to α-KG. The TET enzyme needs to bind to α-KG to play its role. Van Damme M et al^[25]^ found the TET 1 and 3 and IDH2 levels are decreased in Chronic Lymphocytic Leukemia cells compared with those in healthy B cells, suggesting that epigenetic changes based on the TET/IDH function are potentially associated with disease progression. Chunping Du et al^[26]^ also found that the loss of 5-hmC in gastric cancer was mainly correlated with the downregulation of IDH2. Our study found that the IDH2 levels were also reduced in HBV-related HCC tissues and the decrease was positively correlated with the TET1 and 5-hmC. These results suggested that the decline of TET1 is related to the decline of IDH2 and lead to the 5-hmC decrease.

There are also some deficiencies in this study. Firstly, the number of samples was small, which may have a certain impact on the statistical results. Secondly, this study was a cross-sectional study, lacking control corhort and long-term follow-up data of patients. Thirdly, this study did not investigate the intrinsic relationship of the 5-hmC and the TET or IDH. Although further studies are needed to solve these problems, the present study still provides important evidence to disclose the decrease and the mechanism of 5-hmC in tumor tissues of HBV-related HCC patients.

## 5. Conclusion

In summary, our study showed the 5-hmC was decreased in HBV-related HCC tumor tissues. Additionally, the decreased TET protein family, in particular, TET1 was the main factor that reduced the 5-hmC in the tumor tissues of HBV-infected HCC. Furthermore, the reduced IDH2 played an important role in the decline of TET1. These findings disclosed the mechanism of the 5-hmC deficiency which might related to the occurrence and progress of the HBV-related HCC and gave some ideas for the further treatment of the disease.

## Author contributions

**Conceptualization:** Yansheng Jia, Lihong Ye, Erhei Dai.

**Data curation:** Yansheng Jia, Zhiquan Liu, Junhua Feng.

**Investigation:** Zhiquan Liu, Lihong Ye, Haicong Zhang.

**Methodology:** Erhei Dai.

**Software:** Muwei Dai.

**Supervision:** Erhei Dai.

**Writing – review & editing:** Yansheng Jia.
